# Vancomycin-resistance gene cluster, *vanC*, in the gut microbiome of acute leukemia patients undergoing intensive chemotherapy

**DOI:** 10.1371/journal.pone.0223890

**Published:** 2019-10-10

**Authors:** Armin Rashidi, Zhigang Zhu, Thomas Kaiser, Dawn A. Manias, Shernan G. Holtan, Tauseef Ur Rehman, Daniel J. Weisdorf, Alexander Khoruts, Gary M. Dunny, Christopher Staley

**Affiliations:** 1 Division of Hematology, Oncology, and Transplantation, Department of Medicine, University of Minnesota, Minneapolis, MN, United States of America; 2 Department of Surgery, University of Minnesota, Minneapolis, MN, United States of America; 3 BioTechnology Institute, University of Minnesota, St. Paul, MN, United States of America; 4 Department of Microbiology and Immunology, University of Minnesota, Minneapolis, MN, United States of America; 5 Division of Gastroenterology, Hepatology, and Nutrition, Department of Medicine, University of Minnesota, Minneapolis, MN, United States of America; University of Kentucky, UNITED STATES

## Abstract

Two recent reports suggested that the less common, less virulent enterococcal species, *Enterococcus gallinarum* and *E*. *casseliflavus*, with low-level vancomycin resistance due to chromosomally encoded *vanC1* and *vanC2/3*, may influence host immunity. We reported that peri-transplant gut colonization with *E*. *gallinarum* and *E*. *casseliflavus* is associated with lower mortality after allogeneic hematopoietic cell transplantation (HCT). Because most acute leukemia patients undergoing HCT have received intensive chemotherapy (usually requiring prolonged hospitalization) for their underlying disease before HCT, we hypothesized that some may have acquired *vanC*-positive enterococci during chemotherapy. Therefore, we evaluated the presence of the *vanC* gene cluster using *vanC1* and *vanC2/3* qPCR in thrice-weekly collected stool samples from 20 acute leukemia patients undergoing intensive chemotherapy. We found that an unexpectedly large proportion of patients have detectable *vanC1* and *vanC2/3* (15% and 35%, respectively) in at least one stool sample. Comparing qPCR results with 16S rRNA gene sequencing results suggested that *E*. *gallinarum* may reach high abundances, potentially persisting into HCT and influencing transplant outcomes.

## Introduction

Hematopoietic cell transplantation (HCT) is a potentially curative approach for many patients with high-risk hematologic malignancies. After their initial anti-neoplastic treatment, often including intensive chemotherapy, such patients are referred for HCT. Gut colonization with antibiotic-resistant organisms is common during HCT [1–3]. The genus *Enterococcus*, frequently harboring antibiotic resistance genes (ARGs), is one of the most common gut colonizers in HCT patients [4,5]. The plasmid-encoded *vanA* gene is the most common enterococcal ARG conferring high-level vancomycin resistance to *E*. *faecium* and *E*. *faecalis* (VRE), with high transmissibility and propensity to cause major clinical infections [6,7]. In contrast, the *vanC* gene cluster is chromosomally encoded and confers low-level, intrinsic vancomycin resistance to the less commonly encountered *E*. *casseliflavus* (*vanC2/3* gene) and *E*. *gallinarum* (*vanC1* gene) [8,9].

We recently reported that colonization with *E*. *casseliflavus* or *E*. *gallinarum* is associated with lower transplant-related mortality and better survival [10]. The mechanisms underlying this association are unknown, but alterations in host immunity have been reported [11]. In addition, it is not known whether patients acquire *E*. *casseliflavus* and *E*. *gallinarum* during HCT or before HCT during the treatment of their underlying hematologic disorder. To determine when these species are acquired, we investigated the presence of *vanC* genes in the stool of acute leukemia patients undergoing intensive chemotherapy.

## Methods

### Patients and samples

We collected thrice-weekly stool samples between hospital admission and day 28 of chemotherapy (or discharge) from 20 acute leukemia patients receiving intensive chemotherapy. Day 0 was defined as the first day of chemotherapy. Our Institutional Review Board at the University of Minnesota approved the protocol (ID #2017NTLS052) and all patients provided written informed consent. Details of the protocol have been reported elsewhere [12]. Patients remained in private rooms for their entire hospitalization. Contact isolation was implemented if colonization or clinical infection with any vancomycin-resistant enterococci (including *E*. *gallinarum* or *E*. *casseliflavus*) was identified. We defined intensive chemotherapy as any regimen requiring a planned hospitalization of approximately 4 weeks. Patients did not enroll to the same study upon relapse. At our institution, we use levofloxacin for antibacterial, acyclovir for antiviral, and an azole for antifungal prophylaxis starting with chemotherapy until neutrophil recovery, and cefepime as the initial empiric antibiotic for neutropenic fever. Variations to this recommendation were allowed.

### DNA extraction

Samples were stored at -80°C prior to DNA extraction. DNA was extracted from thawed fecal samples using the DNeasy® PowerSoil® kit (Qiagen, Hilden, Germany). Approximately 0.25 g fecal sample were added to the PowerBead tube and processed according to the manufacturer’s instructions using the automated QIAcube platform with the inhibitor removal technology protocol. A final volume of 100 μl DNA solution was obtained and the concentration of extracted DNA was quantified with a Qubit^TM^ 4 fluorometer and dsDNA HS Assay kit (Thermo Fisher Scientific, Waltham, MA, USA).

### 16S rRNA amplicon sequencing

The V4 hypervariable region of the 16S rRNA gene was sequenced on the Illumina MiSeq platform (Illumina Inc., San Diego, CA, USA). Raw sequence data are available under NCBI BioProject #SRP141394. Sequence data were processed as previously described [13]. Briefly, sequences were paired-end joined, trimmed for quality, and aligned against the SILVA database version 132 [14]. Sequences were subjected to a 2% pre-cluster step to remove likely errors [15], and chimeras were identified and removed using UCHIME ver. 4.2.40 [16]. Operational taxonomic units were classified at 97% sequence similarity using the furthest-neighbor algorithm, and taxonomic classifications were made against the version 16 release from the Ribosomal Database Project [17].

### Quantitative PCR (qPCR)

qPCR assays targeting the vancomycin resistance genes *vanC1* and *vanC2/3* were used as described previously (**Table 1**) [18]. The primers were synthesized by Integrated DNA Technologies (IDT; Coralville, IA, USA) and diluted to 10 μM working stocks. Primer targets were cloned into a gBlocks plasmid standard (IDT), using previously published accession numbers [18]. Standard curves ranging from 10^6^ to 10^1^ gene copies were prepared from 10-fold serial dilutions of the plasmid standard. Each standard concentration was run in triplicate on each plate. Two negative (sterile water) controls were included on each plate. PCR reactions included 10 μl QuantiTect SYBR Green MasterMix (Qiagen), 1 μl of each primer (1.5 μl for *vanC1*), 6 μl nuclease-free water, and 2 μl DNA template. PCR was carried out using the LightCycler 480 II thermocycler (Roche, Basel, Switzerland) with the following two-step thermocycling conditions for vancomycin resistance genes: an initial denaturation at 95°C for 15 min, followed by 40 cycles of 95°C for 15 s and 60°C for 1 min. DNA from all samples was run in duplicate. Gene copies were extrapolated from the standard curve using the absolute quantification/2^nd^ derivative max method with the LightCycler 480 software, for which the manufacturer recommends that error should be <0.2 for accurate quantitation. Data were reported as gene copies per ng DNA if both replicates showed quantifiable amplification. If only one replicate was quantifiable, results were reported as positive, but not quantifiable.

**Table 1:** Primers and performance characteristics for qPCR assays.

**Target gene**	Primer	Sequence (5’-3’)	Size (bp)	T_m_* (°C)	Efficiency (%)	Error
*vanC1*	VanC1-F	TGCTTGTGATGCGATTTCTC	204	83.57 ± 0.07	91.44 ± 1.41	0.05 ± 0.03
	VanC1-R	ATCGCTCCTTGATGGTGAC				
*vanC2/3*	VanC23-F	GGGAAGATGGCAGTATCCAA	102	80.34 ± 0.09	94.81 ± 3.09	0.04 ± 0.03
	VanC23-R	GCAGCAGCCATTTGTTCATA				

*Melting temperature (T_m_), shown as mean ± standard error based on standard curves generated in this study.

### Statistical analysis

Alpha diversity was measured by Shannon index, incorporating both richness and evenness of the abundances of bacterial community members [19]. We determined the correlation between *Enterococcus* relative abundance and *vanC* abundance by a Spearman’s correlation test.

## Results

**Fig 1:**
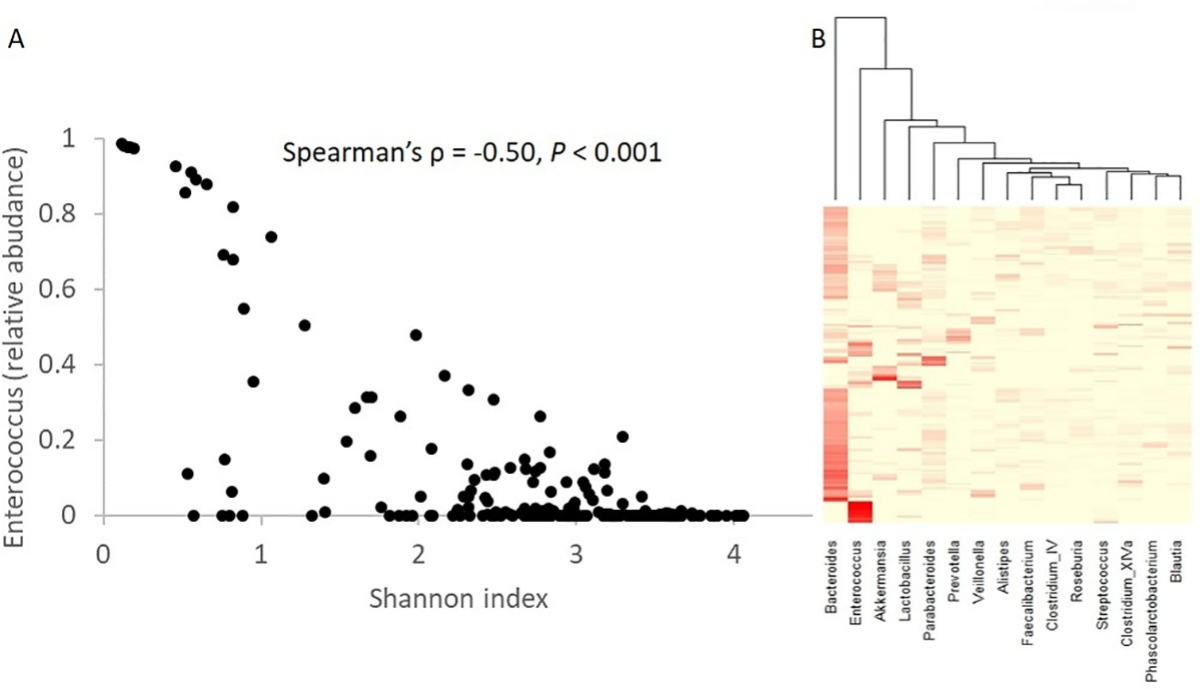
Microbiota diversity and composition. There is a negative correlation between Shannon diversity and *Enterococcus* relative abundance. Each point represents a sample. **(B)** Heatmap of microbiota relative abundances from 16S rRNA amplicon sequencing. Each column shows a genus and each row represents a sample. Only the 15 most abundant genera are shown.

**Fig 2:**
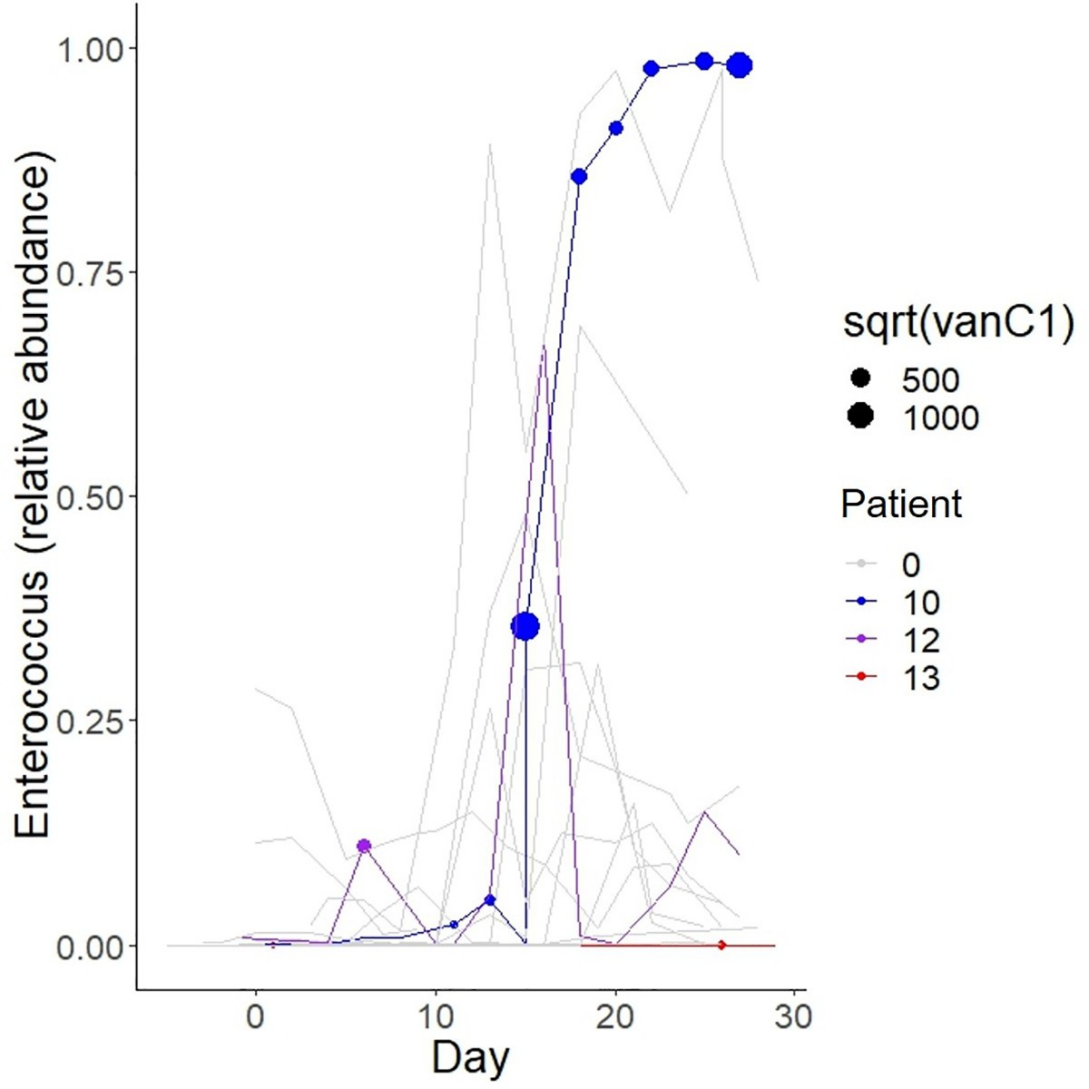
Changes in *Enterococcus* relative abundance and *vanC1* copies per ng DNA over time. Each line represents samples from the same patient. Patients with detectable *vanC1* (patients #10, 12, and 13) are shown in color while all other patients (labeled as #0) are grayed out for improved visibility. *vanC1*-positive samples are indicated with a circle, with a diameter proportional to the square root of *vanC1* gene copies per ng DNA. Day 0 is the first day of chemotherapy.

Detailed patient characteristics are shown in **Table 2**. We collected and analyzed 207 stool samples. There was a negative correlation between Shannon diversity and *Enterococcus* relative abundance (Spearman’s ρ = -0.50, *P* < 0.001; **Fig 1A**). **Fig 1B** shows the heatmap of the 15 most common genera among all samples. *Enterococcus* was the second most common genus, with an aggregate mean (range) relative abundance of 10 (0–99)%, following *Bacteroides*, with an aggregate mean (range) relative abundance of 25 (0–92)%. **Fig 2** shows changes in *Enterococcus* relative abundance and *vanC* gene copies per ng DNA over time for each patient. *vanC1* was detectable in 11 (5%) samples from 3 (15%) patients, with a median of 30,147 (range: 2–1,873,333) copies per ng DNA. *vanC1* was quantifiable in all positive samples. *vanC2/3* was detectable in 19 (9%) samples from 7 (35%) patients, with a median of 13 (range: 1–232) copies per ng DNA in quantifiable cases (13 samples). All three patients with *vanC1* also had detectable *vanC2/3*.

**Table 2:** Patient, disease, and treatment characteristics.

Patient	Age (y), Sex	Disease, Phase	Prior intensive chemotherapy	Interval from last intensive chemotherapy	Chemotherapy during study
1	22, M	AML, Induction	-	-	7+3
2	60, M	AML, Re-induction	Yes	0.5 months	MEC
3	68, F	AML, Re-induction	Yes	7 months	Clo/Ara-C
4	36, F	ALL, Induction	-	-	GRAAL [20]
5	52, F	ALL, Induction	-	-	PETHEMA [21]
6	35, M	AML, Re-induction	Yes	7 months	Clo/Ara-C
7	67, F	AML, Induction	-	-	7+3
8	74, F	AML, Induction	-	-	7+3
9	58, F	AML, Induction	-	-	7+3
10	50, F	AML, Re-induction	Yes	6 months	MEC
11	65, M	AML, Re-induction	Yes	4 months	MEC
12	73, M	AML, Induction	-	-	7+3
13	51, F	AML, Induction	-	-	7+3
14	53, M	ALL, Induction	-	-	GRAAL
15	23, M	ALL, Induction	-	-	PETHEMA
16	52, F	AML, Induction	-	-	7+3
17	52, F	AML, Re-induction	Yes	10 months	MEC
18	68, M	AML, Induction	-	-	7+3
19	22, M	AML, Re-induction	Yes	4 months	MEC
20	61, F	AML, Induction	-	-	7+3

7+3: Idarubicin + Cytarabine; ALL: Acute lymphoblastic leukemia; AML: Acute myeloid leukemia; Clo/Ara-C: Clofarabine + Cytarabine; F: Female; M: Male; MEC: Mitoxantrone + Etoposide + Cytarabine

We classified patients into 3 groups (**Table 3**). Group A (3 patients) had at least one *vanC1*-positive sample; group B (4 patients) had at least one *vanC2/3*-positive sample, but no *vanC1*-positive sample; group C (13 patients) had no *vanC1*- or *vanC2/3*-positive samples. No relationship was found between antibiotic exposure and the *vanC* group, though this observation was limited by the small sample size and high variability of antibiotic exposures. Thirteen patients received induction and seven received re-induction/salvage chemotherapy. Treatment phase was not associated with the *vanC* group (*P* = 0.80, Fisher’s exact test).

**Table 3:** *vanC* group, antibiotics, and clinical outcomes.

**Pt**	Group^1^	Pre-and post-*vanC* antibacterial antibiotics^2^	NF	BSI	CDI	Relapse/ Progression	Survival
1	B	Pre: Cefepime, Pip-Tazo Post: Cefepime, Pip-Tazo, Vanc	D14	-	D9	-	Alive, 22m
2	B	Pre: Cefepime, FQN, Pip-Tazo, Vanc Post: Cefepime, Daptomycin	D8	D14 (VRE)	-	13m	Alive, 22m
3	C	FQN, Pip-Tazo	D7	-	-	0.5m	Dead, 0.5m
4	C	Cephalexin, FQN	-	-	-	-	Dead, 22m
5	C	Cefepime, Ceftriaxone, Vanc	D26	D26 (*Strep*. *mitis*)	-	-	Alive, 19m
6	B	Pre: FQN, Meropenem Post: Aztreonam, Daptomycin, FQN, Linezolid, Metronidazole, Vancomycin	D13	D14 (*Strep*. *sanguinis*)	-	7m	Dead, 12m
7	C	FQN, Cefepime, Metronidazole	D8	-	D7	9m	Dead, 20m
8	C	FQN, Cefepime, Vanc	D13	D13 (MRSA)	-	8m	Dead, 11m
9	B	Pre: FQN, Cefepime, Meropenem, Pip-Tazo Post: FQN, Meropenem, Metronidazole	D5	-	-	8m	Dead, 9m
10	A	Pre: FQN, Cefepime Post: Azithromycin, Cefepime, FQN, Metronidazole, Vanc	D10	-	-	13m	Dead, 20m
11	C	FQN, Cefepime, Ertapenem, Metronidazole, Pip-Tazo	D7	-	-	1m	Dead, 2m
12	A	Pre: Cefdinir Post: Azithromycin, Cefdinir, Cefepime, FQN, Metronidazole, Pip-Tazo, Vanc	D10	-	D10	5m	Dead, 13m
13	A	Pre: Cefdinir, Cefepime, FQN, Metronidazole, Vanc Post: Cefdinir, Cefepime, Doxycycline, Vanc	D7	-	-	8m	Dead, 11m
14	C	FQN	-	-	-	-	Alive, 19m
15	C	FQN, Cefepime, Pip-Tazo, Vanc	D-1	D-1 (*Strep*. *mitis*)	-	-	Alive, 19m
16	C	FQN, Cefepime, Meropenem, Vanc	D-3	-	-	1m	Dead, 4m
17	C	FQN, Cefepime, Pip-Tazo, Vanc	D9	-	-	5m	Dead, 12m
18	C	FQN, Cefepime	D15	-	-	8m	Dead, 15m
19	C	FQN, Pip-Tazo, Vanc	D9	D9 (*Strep*. *mitis*)	-	1m	Dead, 6m
20	C	Cefepime, Clindamycin, FQN, Metronidazole, Nitrofurantoin, Vancomycin	D5	-	D12	5m	Dead, 5m

^1^Groups: (*i*) group A, ≥1 *vanC1*-positive sample, (*ii*) group B, ≥1 *vanC2/3*-positive but no *vanC1*-positive sample, and (*iii*) group C, no *vanC1*- or *vanC2/3*-positive samples.

^2^For patients with at least one *vanC*-positive sample (groups A and B), pre and post antibiotics refer to those initiated before and after the first positive sample. For patients without any *vanC*-positive samples (group C), antibiotics during chemotherapy are shown. BSI: Bloodstream infection; CDI: *Clostridium difficile* infection; FQN: Fluoroquinolone; m: months; MRSA: Methicillin-resistant *Staphylococcus aureus*; NF: Neutropenic fever; Pip-Tazo: Piperacillin-tazobactam; Pt: Patient; Vanc: Vancomycin; VRE: Vancomycin-resistant *E*. *faecium*

We next evaluated whether *vanC* gene abundance correlates with *Enterococcus* relative abundance as determined by 16S rRNA gene amplicon sequencing. For *vanC1*, this correlation was strong (Spearman’s ρ = 0.78, *P* = 0.004) but there was no correlation between *vanC2/3* and *Enterococcus* relative abundance (ρ = 0.31, *P* = 0.30). Similarly, *vanC1* correlated negatively with Shannon diversity (ρ = -0.64, *P* = 0.04), but *vanC2/3* did not (ρ = 0.23, *P* = 0.41). The primary species known to harbor *vanC2/3* is *E*. *casseliflavus*; therefore, these results suggested that *E*. *casseliflavus* comprised only a minor proportion of enterococci in *vanC2/3*-positive samples, hence its relative abundance (expected to positively correlate with *vanC2/3* gene abundance) did not substantially influence the relative abundance of all enterococci combined. Variations in *Enterococcus* relative abundance in these samples were likely largely driven by *E*. *faecium* and *E*. *faecalis*. In contrast, the strong correlation between *vanC1* gene abundance, primarily present in *E*. *gallinarum*, and *Enterococcus* relative abundance suggested that *E*. *gallinarum* was an abundant species in *vanC1*-positive samples.

We next focused on the three patients whose stools had detectable *vanC1*. The first stool sample with detectable *vanC1* was collected on day 11 (sample 6) in patient #10, day 6 (sample 4) in patient #12, and day 1 (sample 2) in patient #13. These findings suggested that patients #10 and #12 and possibly patient #13 did not have *vanC1* in their stool at baseline but acquired it during hospitalization. Patient #10 continued to have detectable *vanC1* in all stool samples following the first positive sample. Interestingly, *vanC1* was detectable in stool samples collected from this patient during subsequent HCT. Two other patients in our cohort underwent HCT after completing chemotherapy. Patient #1 (with detectable *vanC2/3* during chemotherapy) had detectable *vanC2/3* in stool samples collected during HCT. Patient #14 did not have detectable *vanC1* or *vanC2/3* during chemotherapy or HCT. No patient developed a clinical infection with a *vanC*-positive *Enterococcus* sp. Within the limits of a small sample size, we found no obvious differences in clinical outcomes (**Table 3**).

## Discussion

Gut colonization with *E*. *casseliflavus* and *E*. *gallinarum* is uncommon, occurring in only about 5% of individuals (hospitalized or non-hospitalized) [22,23]. Because of this rarity, expected non-transmissibility of chromosomally encoded *vanC* genes, and overall low pathogenicity, *E*. *casseliflavus* and *E*. *gallinarum* have received little attention in the medical literature. However, two recent studies provided evidence that *E*. *casseliflavus* and *E*. *gallinarum* may influence host immunity. In one study, *E*. *gallinarum* translocated from the gut into the organs and drove autoimmunity in genetically predisposed mice [11]. In the second study, we reported improved survival due to lower transplant-related mortality in allogeneic HCT recipients who had peri-transplant gut colonization with *E*. *casseliflavus* or *E*. *gallinarum* [10]. Because the source of these bacteria in the gut is unknown, we conducted the present study to determine whether patients with acute leukemia (the most common indication for allogeneic HCT) acquire *E*. *casseliflavus* and *E*. *gallinarum* during their ~4-week hospitalization for intensive chemotherapy.

We used *vanC1* and *vanC2/3* as surrogates for *E*. *gallinarum* and *E*. *casseliflavus*, respectively, because with rare exceptions [24,25], these genes are species-specific. Our results suggest a higher than expected incidence of colonization with *E*. *gallinarum* (15% of patients) and *E*. *casseliflavus* (35% of patients) in intensively treated patients with acute leukemia. *vanC1* was detectable at much higher copy numbers than *vanC2/3*. Although we do not know the source of *E*. *gallinarum* and *E*. *casseliflavus*, *vanC* genes were not present in baseline stool samples collected from patients, suggesting that prolonged hospitalization, extensive healthcare contact, major dietary changes during intensive chemotherapy, and microbiota alterations contributed to the acquisition and expansion of *E*. *gallinarum* and *E*. *casseliflavus* to detectable levels. For example, disruptions of gut microbial communities due to antibiotics may have suppressed putative natural competitors of *E*. *gallinarum* and *E*. *casseliflavus*. The negative correlation we found between *vanC1* copy number and Shannon diversity supports this hypothesis. Approximately one third of all *vanC*-positive rectal swabs in a previous study of intensively treated cancer patients tested positive after the first week of hospitalization, supporting the possibility of nosocomial acquisition [26].

Although still controversial [27,28], some previous studies have suggested that antibiotic-induced changes in the gut resistome are transient, with a rapid reversion of ARGs to baseline after discontinuation of antibiotics [29]. Since exposure to antibiotics is nearly universal during chemotherapy, patients receiving re-induction/salvage chemotherapy have prior exposure to antibiotics. This is in contrast to patients receiving induction therapy, who may not have a significant prior history of antibiotic exposure. Our observation of no association between treatment phase and *vanC* detectability argues against permanent changes in the gut resistome due to induction chemotherapy and its associated antibiotics. However, *vanC* genes were detectable during subsequent HCT in stool samples collected from both patients who underwent HCT and had detectable *vanC* during chemotherapy. One possibility is that antibiotic selective pressure is necessary to maintain *vanC*, which was lost after completion of chemotherapy (and discontinuation of antibiotics) but recurred during HCT due to repeated exposure to antibiotics. Another possibility is that *vanC* persisted between completion of chemotherapy and HCT. In the absence of samples between different phases of treatment, we cannot distinguish between these possibilities.

Because chemotherapy-induced gut barrier damage facilitates bacterial translocation, even a transient gut colonization with *vanC*-positive enterococci during chemotherapy could lead to systemic spread of these bacteria, their structural components, or metabolites. This could potentially influence host immunity and transplant outcomes in *vanC*-positive acute leukemia patients who proceed to allogeneic HCT. More research is needed to determine the incidence of persistent low-level bacteremia with *vanC*-positive bacteremia and whether it is mechanistically linked with immune and non-immune effects after HCT.
